# 30 second screening test and education reduce chronic pain incidence after blood donation: Large prospective observational study from Japan

**DOI:** 10.1097/MD.0000000000041491

**Published:** 2025-02-07

**Authors:** Yusuke Hagiwara, Tatsuo Nakamura, Hiroko Shima, Yoshihiko Tani, Yoshimitsu Takahashi, Kentaro Sonoki, Keishichirou Moroi, Ryu Yoshida

**Affiliations:** a Department of Orthopaedic Surgery, Toho Kamagaya Hospital, Chiba, Japan; b Department of General Medicine, Uji-Tokushukai Medical Center, Kyoto, Japan; c Department of Otolaryngology, Kyoto University Hospital, Kyoto, Japan; d Japanese Red Cross Society, Tokyo, Japan; e Department of Health Informatics, Kyoto University School of Public Health, Kyoto, Japan; f Department of Plastic and Reconstructive Surgery, Shizuoka Cancer Center, Shizuoka, Japan; g Department of Anesthesia, Nara Medical University, Nara, Japan; h Department of Orthopaedic Surgery, Cedars Sinai Medical Center, Los Angeles, CA.

**Keywords:** blood draw, blood draw related neuropathic pain, chronic pain, complex regional pain syndrome, nerve injury

## Abstract

Venipuncture is a commonly performed procedure to obtain blood for testing or donation, but nerve injuries with persistent or chronic pain can occasionally occur. We implemented Phalen elbow flexion shoulder abduction (PEFSA) test to screen potential blood donors who may be at risk for persistent or chronic nerve pain after blood donation. Data were prospectively collected on all blood donors at Red Cross centers in Kansai area of Japan for 5 years prior to PEFSA test implementation, and then for 2 years after test implementation. Potential donors who had positive PEFSA test were given a handout informing them of potentially elevated risk for nerve complications. All donors who had persistent pain were followed and treated at Red Cross centers. There were 3,877,975 donors before and 1,461,965 potential donors after PEFSA test implementation. A total of 221 out of 15,776 potential donors with positive PEFSA test decided to stop donation. Prior to test implementation, 98 (0.00253%) donors developed persistent pain. Nineteen had symptoms lasting over 1 year. Six were diagnosed with complex regional pain syndrome (CRPS). After PEFSA test implementation, 22 (0.00151%) developed persistent pain. None had symptoms lasting >1 year or developed CRPS. Our data suggest that the PEFSA test alone do not identify high risk potential donors, but also educating the positive potential donors significantly decrease incidence of persistent pain and CRPS. To our knowledge, this study is the first to test an intervention that can help prevent persistent pain or CRPS from blood donation.

## 1. Introduction

Venipuncture is a commonly performed invasive procedure to obtain blood for testing or donation. While it is a safe, routine procedure, complications can occasionally occur. During the percutaneous insertion of the needle, cutaneous nerves can be injured. The reported incidence of nerve injuries from venipuncture range from 1:25,000^[1]^ and 1:21,000^[2]^ to 1:6929.^[3]^ In a recent study examining more than 1 million venipunctures, 16 out of 1082,053 individuals were diagnosed with obvious nerve injuries.^[4]^ Neuropathic pain from blood draw typically resolves quickly but can sometimes persist for months to years. The symptoms can be quite severe, sometimes resembling those of complex regional pain syndrome (CRPS).^[5–7]^ Financial implications of such complication can be significant for medical facilities. One patient who had nerve injury after blood draw was awarded 2.5 million dollars by court.^[8]^

Horowitz studied anatomic relationships of cutaneous nerves and veins and found that they run intimately together, with nerves frequently running over or intertwining with veins.^[6]^ He noted that needle injury from venipuncture must therefore be frequent, yet the actual incidence of chronic pain from venipuncture is infrequent. Therefore, he concluded that other factors must be present for patients to develop chronic pain from venipuncture.

The presence of compressive neuropathy may be such a factor. We have previously reported a series of 10 blood donors with persistent pain after venipuncture. Two patients had spontaneous resolution of pain, but 8 patients were found to have compressive neuropathy (carpal tunnel syndrome and/or cubital tunnel syndrome). All 8 patients had resolution of pain with treatment of compressive neuropathy.^[9]^

To quickly identify undiagnosed compressive neuropathies, we developed a simple 30 second test. We hypothesized that the test would identify donors at risk and help prevent development of chronic pain after blood donation. To test our hypothesis, we performed a large multicenter study on blood donors over a 7-year period. We prospectively collected data before and after implementation of the Phalen elbow flexion shoulder abduction (PEFSA) test at all Red Cross centers in the Kansai area of Japan. Here, we present the results and finding from the study.

## 2. Materials and methods

The study was conducted at Japanese Red Cross centers in the Kansai area. Ethical review board approval was obtained from the Japanese Red Cross Society for Kansai area on March 19, 2013.

Blood donation was declined from those with 1. age < 16 years or >69 years, 2. body weight < 45 kg for males and <40 kg for females, 3. systolic blood pressure < 90 mm Hg or >180 mm Hg, 4. heart rate < 40 beats/min, 5. specific gravity of blood < 1.052 or hemoglobin < 12 g/dL, 6. pregnant, 7. fever, 8. heart disease (including heart attack, angina, myocarditis, valvular disease, and arrythmia) or blood disorder (including hemophilia, purpura, aplastic anemia, leukemia, polycythemia vera, and significant anemia).

**Figure 1.**
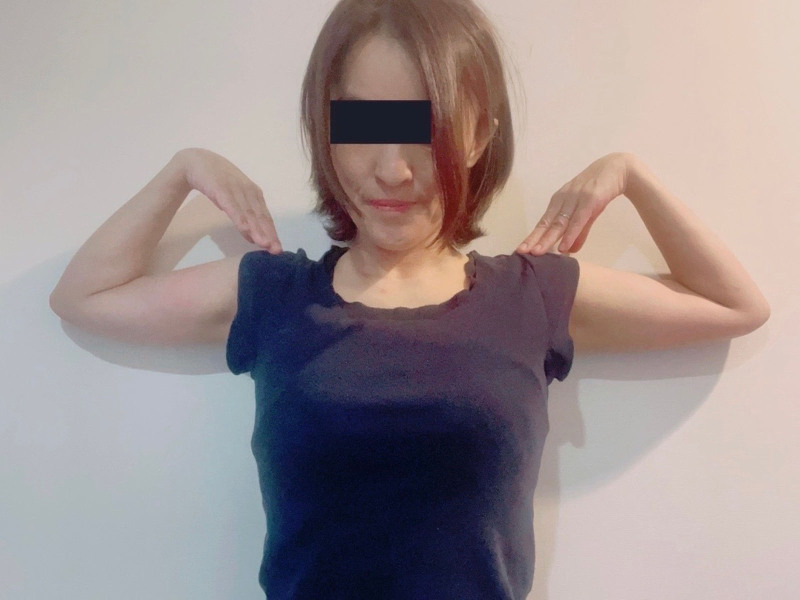
Demonstration of Phalen elbow flexion shoulder abduction (PEFSA) test. The shoulders are abducted 90 degrees, elbows are maximally flexed, and wrists are maximally flexed. Individuals are instructed to hold this position for 30 seconds. Consent was obtained from the patient for publication of this photo.

After screening with the above criteria, potential donors from September 1, 2008 to August 31, 2013 (period prior to PEFSA test implementation) proceeded to donate blood. Potential donors after PEFSA test implementation (from September 1, 2013 to August 31, 2015) performed the PEFSA test prior to donation under the supervision of Japanese Red Cross staff. They were instructed to abduct their shoulders 90 degrees, maximally flex the elbows, and maximally flex their wrists for 30 seconds (Fig. 1). The results of the test were classified into 3 grades: A – no discomfort, B – experienced discomfort but was able to maintain position for 30 seconds, C – unable to maintain position for 30 seconds. The potential donors who were grade B or C were given a handout (Data S1, Supplemental Digital Content, http://links.lww.com/MD/O367). The handout informed the potential donors that they may be at increased risk for nerve complications from donation including chronic pain and that they may have undiagnosed compression neuropathy such as carpal tunnel syndrome or cubital tunnel syndrome. If they still desired, the potential donors then proceeded to consenting and donating blood. All donors were instructed to contact the blood center if they had persistent pain after donation.

All donors who developed persistent pain after blood donation were followed and treated at their local Japanese Red Cross center. Presence of Tinel sign was assessed and recorded. Diagnosis of CRPS was made based on the criteria published by the Japanese Ministry of Health, Labour and Welfare CRPS Research Group.^[10]^ The diagnosis requires at least 2 symptoms reported by patients (atrophy of skin, nail, or hair; limitation in range of motion; continuous or disproportional pain or hypersensitivity; increase or decrease in perspiration; or edema) and 2 exam findings (atrophy of skin, nail, or hair; limitation in range of motion; allodynia; increase or decrease in perspiration; edema).

We analyzed the data using Fisher exact test. All analyses were performed using R version 3.3.3 (The R Foundation). *P* values < .05 were considered statistically significant. There was no funding source for this study.

## 3. Results

There were 3,877,975 donors (between September 1, 2008 and August 31, 2013) before and 1461,965 potential donors (between September 1, 2013 and August 31, 2015) after implementation of the PEFSA test. With PEFSA testing, 1,444,758 (98.2%) potential donors were grade A, 13,702 (0.94%) were grade B, and 2074 (0.14%) were grade C. All grade A donors proceeded to donate blood. 196 out of 13,702 (1.43%) grade potential B donors decided not to donate blood. Twenty-five of 2074 (1.21%) grade C potential donors decided not to donate blood.

Prior to implementation of the PEFSA test, 98 out of 3,877,975 (0.00253%) donors developed persistent pain. Seventeen had resolution of symptoms within 4 weeks, 62 had 4 weeks to 1 year of symptoms, and 19 had symptoms lasting over 1 year. Six patients were diagnosed with CRPS.

**Figure 2.**
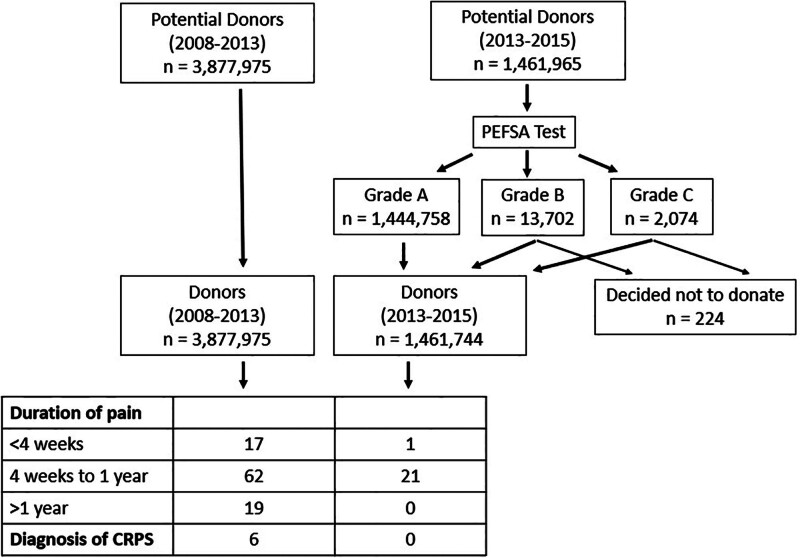
Diagram summarizing outcomes of blood donations before and after Phalen elbow flexion shoulder abduction (PEFSA) test implementation.

After PEFSA test implementation, 22 out of 1,461,741 (0.00151%) donors developed persistent pain. All 22 donors were grade A on PEFSA test. One resolved within 4 weeks, and 21 had 4 weeks to 1 year of symptoms, and none had symptoms lasting >1 year. No donors developed CRPS (Fig. 2).

There was statistically significant difference in rate persistent pain before (98 out of 3,877,975) and after (22 out of 1,461,741) PEFSA test implementation (*P* = .0244).

## 4. Discussion

The PEFSA test was designed as a quick, simple test to screen for compressive neuropathy in the upper extremity. It is a combination of the Phalen test for carpal tunnel syndrome, deep elbow flexion for cubital tunnel syndrome, and shoulder abduction which places additional tension throughout the brachial plexus which gives rise to all nerves of the upper extremity.^[11–13]^ We started implementing the test for blood donors after treating a series of patients with chronic pain after blood donation and noting high incidence of underlying carpal or cubital tunnel syndromes. In our previous pilot study on 296,170 blood donors, the test was able to identify 0.16% donors who had previously undiagnosed compression neuropathy.^[14]^

There are various reports that have shown associations compressive neuropathies and CRPS. In the upper extremity, carpal tunnel syndrome and cubital tunnel syndrome can be underlying causes of CRPS, and surgical nerve decompressions can lead to significant improvement.^[15]^ Successful treatments of CRPS with surgical nerve decompressions have been reported in the lower extremity as well.^[16]^ Additionally, we had previously observed that majority of patients with persistent pain after blood draw had underlying compressive neuropathy.^[9]^ Therefore, we hypothesized that the PEFSA test would help identify potential blood donors at risk for persistent pain.

The results from the current study showed that the PEFSA test did not identify high risk patients. All 22 donors who developed persistent pain were grade A on the PEFSA test. However, significant decrease in the incidence of persistent pain was observed following the implementation of the test. The incidence decreased by 40% from 0.00253% to 0.00151%. There was decrease in all 3 classes of persistent pain (duration <4 weeks, 4 weeks to 1 year, and over 1 year), and most notably, there were no donors who had pain for more than 1 year. No donors met the diagnostic criteria for CRPS. To our knowledge, there are no other interventions described in literature that can decrease pain related complications from blood donation.

One possible reason for the significant decrease is the opting out of donors. If a potential donor was grade B or C, he/she was given a handout explaining possible elevated risk for nerve complications. The potential donor had the opportunity to opt out of blood donation at that time, and some donors (196 from grade B and 25 from grade C) did opt out. This opportunity to opt out may have screened out those who were at high risk to develop persistent pain.

Those who opted out may have had psychological factors putting them at higher risk for chronic pain. There are numerous studies linking psychological factors to development of chronic pain. Fear-avoidance is a theory that tries to explain the development of exaggerated pain perception. The key concept in this theory is that responding to fear of pain by avoidance (instead of confrontation) can lead to maintenance or exacerbation of pain.^[17–19]^ There are also studies showing that a cognitive process called “catastrophizing,” exhibiting exaggerated negativity and assuming worst outcomes, is associated with development of chronic pain.^[20,21]^ Those potential donors who presented for blood donation but opted out after the test may have included a number of people with fear avoidance or catastrophizing behavior.

It is worth noting that potential donors with grade A on the test did not receive the handout. The handout warned that people with positive PEFSA test (grade B or C) may be at increased risk for nerve complications and chronic pain. It would be interesting in future studies to see if a handout describing risks in a patient group (PEFSA positive) would affect those not in the group (PEFSA negative).

We suspect that the most likely reason all 22 patients with chronic pain were negative PEFSA was because there were many more PEFSA-negative donors than PEFSA-positive donors (1,461,744 vs 15,776). The rates of chronic pain PEFSA-negative vs PEFSA-positive donors were close (0.0016% vs 0%). Another possible explanation is that the test reduced long-term chronic pain but not short-term pain because the latter is not necessarily related to underlying compressive neuropathy. Patients without compressive neuropathy can get some short-term pain but are perhaps unlikely to get long-term pain.

The study has several limitations. The subjects were not randomized in this study. However, the data were collected prospectively, and there was no change in any other aspects of blood donation protocol before and after PEFSA test implementation. Therefore, the pre-implementation group was an appropriate control group. Another limitation of the study is that it does not provide explanation of the mechanism. Further studies looking more closely at donors developing chronic pain may help advance our understanding of the mechanism.

## 5. Conclusion

This large multicenter study showed that a quick 30 second test requiring no equipment can reduce the incidence of persistent pain after blood donation by 40%. While additional studies are needed to understand the pathophysiology and prevention mechanism of persistent pain after blood donation, the PEFSA test and patient education had a significant effect on reducing incidence of chronic pain and CRPS in blood donors.

## Acknowledgments

Dr Yuji Inada developed the PEFSA test and played an integral role in this research project. He sadly passed away in February 2020.

## Author contributions

**Conceptualization:** Yusuke Hagiwara, Tatsuo Nakamura, Hiroko Shima, Yoshihiko Tani.

**Data curation:** Yusuke Hagiwara, Tatsuo Nakamura, Hiroko Shima, Yoshihiko Tani.

**Formal analysis:** Yusuke Hagiwara, Tatsuo Nakamura, Hiroko Shima, Yoshihiko Tani, Yoshimitsu Takahashi, Kentaro Sonoki, Keishichirou Moroi, Ryu Yoshida.

**Writing – original draft:** Yusuke Hagiwara, Tatsuo Nakamura, Hiroko Shima, Yoshihiko Tani, Yoshimitsu Takahashi, Kentaro Sonoki, Keishichirou Moroi, Ryu Yoshida.

**Writing – review & editing:** Yusuke Hagiwara, Tatsuo Nakamura, Hiroko Shima, Yoshihiko Tani, Yoshimitsu Takahashi, Kentaro Sonoki, Keishichirou Moroi, Ryu Yoshida.

## Supplementary Material


