# Supplementary material for: 30 second screening test and education reduce chronic pain incidence after blood donation: Large prospective observational study from Japan
Source: Medicine (Baltimore). 2025 Feb 7;104(6):e41491. doi: 10.1097/MD.0000000000041491 (PMC11813037; doi:10.1097/MD.0000000000041491)
Supplement: Supplementary file 1 [file medi-104-e41491-s001.docx]

What is PEFSA test?

-It is a quick 30 second test to test the health of your nerves in the upper limb.

How do you do it?

-Raise both of your arms to the side to 90 degrees. Fully bend your elbows and wrist inwards. Try to hold this position for 30 seconds. Are you able to hold this position for 30 seconds? Are you getting symptoms such as pain, numbness, or tingling?


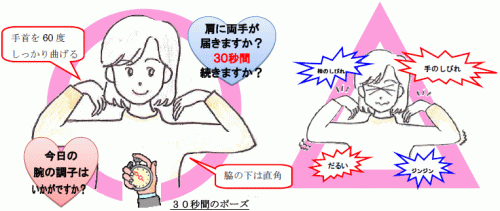


Why might you get such symptoms?

-You may have underlying nerve problems such as carpal tunnel syndrome or cubital tunnel syndrome. People to have nerve problems that are not recognized because they do not experience symptoms with regular daily activities. Presence of nerve problems may increase risk of persistent pain after blood draws.
